# Supplementary material for: The Contribution of Pharmacogenetic Drug Interactions to 90-Day Hospital Readmissions: Preliminary Results from a Real-World Healthcare System
Source: J Pers Med. 2021 Nov 23;11(12):1242. doi: 10.3390/jpm11121242 (PMC8705172; doi:10.3390/jpm11121242)
Supplement: Supplementary file 1 [file jpm-11-01242-s001.zip › jpm-1453490-supplementary.pdf]

Supplemental Material

Table S1. List of Medication Orders/Administration from 12/01/2009 to 12/31/2020 with Orders 30 days Prior to Inpatient Admissions

	**Readmission within 90 days of inpatient admission discharge date in Encounter Fact between 1/1/2010 and 3/31/2021 inclusive**		
**CPIC medications**	**Overall**	**No**	**Yes**
**N (%)**	**2,211 (100)**	**1,935 (87.5)**	**276 (12.5)**
Amitriptyline	26 (1.2)	14 (0.7)	12 (4.3)
Atazanavir	0 (0)	0 (0)	0 (0)
Atomoxetine	5 (0.2)	3 (0.2)	2 (0.7)
Azathioprine	9 (0.4)	5 (0.3)	4 (1.4)
Capecitabine	0 (0)	0 (0)	0 (0)
Celecoxib	287 (13)	238 (12.3)	49 (17.8)
Citalopram	24 (1.1)	18 (0.9)	6 (2.2)
Clomipramine	2 (0.1)	2 (0.1)	0 (0)
Clopidogrel	55 (2.5)	36 (1.9)	19 (6.9)
Codeine	64 (2.9)	45 (2.3)	19 (6.9)
Desipramine	0 (0)	0 (0)	0 (0)
Doxepin	4 (0.2)	0 (0)	4 (1.4)
Efavirenz	0 (0)	0 (0)	0 (0)
Escitalopram	120 (5.4)	93 (4.8)	27 (9.8)
Fluorouracil	4 (0.2)	2 (0.1)	2 (0.7)
Flurbiprofen	0 (0)	0 (0)	0 (0)
Fluvoxamine	1 (0)	1 (0.1)	0 (0)
Fosphenytoin	4 (0.2)	0 (0)	4 (1.4)
Ibuprofen	1064 (48.1)	963 (49.8)	101 (36.6)
Imipramine	0 (0)	0 (0)	0 (0)
Lansoprazole	31 (1.4)	19 (1)	12 (4.3)
Lornoxicam	0 (0)	0 (0)	0 (0)
Meloxicam	35 (1.6)	29 (1.5)	6 (2.2)
Mercaptopurine	1 (0)	1 (0.1)	0 (0)
Nortriptyline	20 (0.9)	15 (0.8)	5 (1.8)
Omeprazole	541 (24.5)	416 (21.5)	125 (45.3)
Ondansetron	2026 (91.6)	1769 (91.4)	257 (93.1)
Pantoprazole	298 (13.5)	204 (10.5)	94 (34.1)
Paroxetine	24 (1.1)	16 (0.8)	8 (2.9)
Peginterferon-Alfa-2a	0 (0)	0 (0)	0 (0)
Peginterferon-Alfa-2b	0 (0)	0 (0)	0 (0)
Phenytoin	6 (0.3)	3 (0.2)	3 (1.1)
Piroxicam	0 (0)	0 (0)	0 (0)
Sertraline	102 (4.6)	84 (4.3)	18 (6.5)
Simvastatin	91 (4.1)	66 (3.4)	25 (9.1)
Tacrolimus	10 (0.5)	5 (0.3)	5 (1.8)
Tamoxifen	6 (0.3)	6 (0.3)	0 (0)
Tenoxicam	0 (0)	0 (0)	0 (0)

Thioguanine	0 (0)	0 (0)	0 (0)
Tramadol	347 (15.7)	252 (13)	95 (34.4)
Trimipramine	0 (0)	0 (0)	0 (0)
Tropisetron	0 (0)	0 (0)	0 (0)
Voriconazole	0 (0)	0 (0)	0 (0)
Warfarin	237 (10.7)	185 (9.6)	52 (18.8)

Table S2. Frequency of 90-day Readmission Diagnostic Categories (N=281)

Diagnostic Categories	Frequency	Percent
Behavioral/Psychiatric	27	9.82
Cancer/Neoplasm	32	11.64
Cardiovascular	16	5.82
Developmental/Disability	1	0.36
Endocrine/Metabolic	48	17.45
Gastrointestinal	30	10.91
Genitourinary	3	1.09
Gynecological	1	0.36
Hematological	16	5.82
Infection/Abscess	2	0.73
Infection/Abscess/Other	9	3.27
Infection/Dermatological/Abscess	3	1.09
Infection/Gastrointestinal	9	3.27
Infection/Genitourinary	3	1.09
Infection/Respiratory	5	1.82
Inflammatory/Rheumatological	2	0.73
Neurological	10	3.64
Obstetrical	21	7.64
Orthopedic/Musculoskeletal	13	4.73
Pain	4	1.45
Pulmonary	10	3.64
Vascular	1	0.36
Vascular/Non-Cardiac	9	3.27

*6 patients missing readmission diagnosis.
